# Micron‐resolution Imaging of Cortical Bone under 14 T Ultrahigh Magnetic Field

**DOI:** 10.1002/advs.202300959

**Published:** 2023-06-20

**Authors:** Tian He, Zhenfeng Pang, Yu Yin, Huadong Xue, Yichuan Pang, Haixin Song, Jianhua Li, Ruiliang Bai, An Qin, Xueqian Kong

**Affiliations:** ^1^ Department of Chemistry Zhejiang University Hangzhou 310027 China; ^2^ Department of Rehabilitation Sir Run Run Shaw Hospital College of Medicine Zhejiang University Hangzhou 310016 China; ^3^ Shanghai Key Laboratory of Orthopedic Implants Department of Orthopaedics Shanghai Ninth People's Hospital Shanghai Jiao Tong University School of Medicine Shanghai 200011 China; ^4^ Interdisciplinary Institute of Neuroscience and Technology (ZIINT) College of Biomedical Engineering and Instrument Science Zhejiang University Hangzhou 310027 China; ^5^ School of Medicine Zhejiang University Hangzhou 310058 China; ^6^ Institute of Translational Medicine Shanghai Jiaotong University Shanghai 200240 China

**Keywords:** cortical bone, magnetic resonance imaging, relaxation, ultrahigh field, ultrashort echo

## Abstract

Compact, mineralized cortical bone tissues are often concealed on magnetic resonance (MR) images. Recent development of MR instruments and pulse techniques has yielded significant advances in acquiring anatomical and physiological information from cortical bone despite its poor ^1^H signals. This work demonstrates the first MR research on cortical bones under an ultrahigh magnetic field of 14 T. The ^1^H signals of different mammalian species exhibit multi‐exponential decays of three characteristic T_2_ or T_2_* values: 0.1–0.5 ms, 1–4 ms, and 4–8 ms. Systematic sample comparisons attribute these T_2_/T_2_* value ranges to collagen‐bound water, pore water, and lipids, respectively. Ultrashort echo time (UTE) imaging under 14 T yielded spatial resolutions of 20–80 microns, which resolves the 3D anatomy of the Haversian canals. The T_2_* relaxation characteristics further allow spatial classifications of collagen, pore water and lipids in human specimens. The study achieves a record of the spatial resolution for MR imaging in bone and shows that ultrahigh‐field MR has the unique ability to differentiate the soft and organic compartments in bone tissues.

## Graphical abstract

Ultrahigh‐field magnetic resonance imaging (MRI) reveals the micron‐level canal structures in cortical bones. The analysis of multi‐exponential relaxation allows spatial separation of collagen, pore, and lipids. Ultrahigh‐field MRI demonstrates the unique ability to distinguish soft and organic compartments in bone tissues.

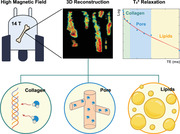

## 1 Introduction

Bones are mineralized biological tissues that support the muscles, enable locomotive functions, and balance the calcium and phosphate in the circulatory system.^[^ ^1^, ^2^ ^]^ Dense and compact cortical tissues are the main contributors to the mechanical strength of bone.^[^ ^3^, ^4^ ^]^ Cortical bones have hierarchical structures that consist of a matrix cemented by inorganic calcium phosphates and organic collagen and of an interconnected network made of water canals and cell chambers (i.e., Haversian–Volkmann canals and lacuna–canaliculi systems).^[^ ^5^, ^6^ ^]^ While the matrix can be considered solid walls, the water canals are the lifeline passages that support live osteocytes in the chambers.^[^ ^7^, ^8^, ^9^ ^]^ Many studies are focused on the nanoscale construction of the matrix^[^ ^10^, ^11^, ^12^, ^13^, ^14^ ^]^ and the microscale morphology of the canals.^[^ ^15^, ^16^, ^17^, ^18^ ^]^ However, the fluids (mostly water) flowing inside the canals carry nutrients and metabolites and transduce signals in and out of the living bone tissue.^[^ ^5^, ^8^, ^19^ ^]^ The flowing water supports the critical physiological functions and biological activities of the bone. Other than nuclear magnetic resonance (MR) spectroscopy (MRS) and MR imaging (MRI), few techniques provide direct information on the water inside cortical bones. For example, ^1^H MR can discern free water from collagen‐bound water via their different relaxation behaviors,^[^ ^20^, ^21^, ^22^, ^23^ ^]^ correlate relaxation characteristics to bone porosity and mechanical properties,^[^ ^23^, ^24^, ^25^, ^26^ ^]^ measure the water diffusivity inside Haversian canals,^[^ ^27^, ^28^, ^29^ ^]^ and be used in vitro and in vivo to characterize bones with different diseases.^[^ ^30^, ^31^, ^32^ ^]^

However, several challenges inhibit the application of MRI to cortical bones. First, the signal‐to‐noise (S/N) ratio of ^1^H MR of bone is poor owing to the low water concentration and short transverse relaxation (T_2_ and T_2_*).^[^ ^31^ ^]^ Second, the relaxation behavior of water in bone is complex and can be affected by the local environment (in the canal or chamber) or by the fluidic state (bound or free).^[^ ^20^, ^22^, ^30^, ^33^, ^34^ ^]^ Thus, water signals in bones are difficult to acquire and interpret. To date, few consider MRI to be an adequate imaging tool for microscopic studies of cortical bones owing to its poor resolution at the submillimeter level.^[^ ^35^ ^]^

Here, we look beyond the common 1–3 T magnetic fields and analyze the cortical bone under an ultrahigh magnetic field of 14 T. Under 14 T, the ^1^H T_2_ and T_2_* relaxations of the cortical bone in mammals (including rat, sheep, swine, bovine, and human) exhibit multiple distributions. Using MR spectroscopy and comparative sample treatments, we identify these components as collagen‐bound water, pore water, and lipids, respectively. We further perform MRI on cortical bones using the ultrashort echo time (UTE) pulse sequence.^[^ ^36^, ^37^ ^]^ The boosted signal‐to‐noise ratio under 14 T and the clear relaxation characteristics enable an unprecedented micron‐scale resolution of the cortical bone histology and anatomy.

## 2 Results

**Figure 1:**
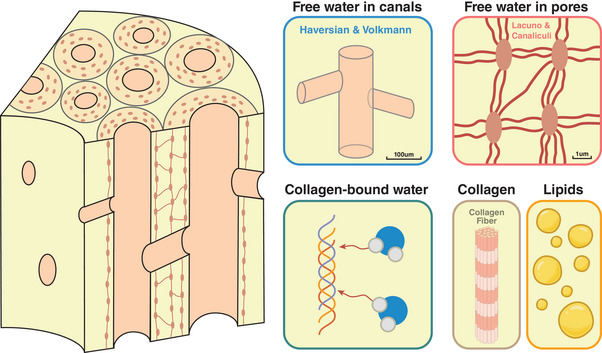
Anatomy and composition of cortical bone. Water flows inside the cannular microstructures of the Haversian and Volkmann canals (≈100 µm for human bone) and the lacuno‐canalicular network (≈1 µm). A considerable amount of water is associated with the collagen fibers via hydrogen bonds. The collagen fibers and lipids also contribute to the ^1^H MR signal.

The ^1^H MR signals of cortical bones arise from several major sources (**Figure** **1**): 1) the free‐flowing water in the Haversian canals, 2) the water in the lacuno‐canalicular system, 3) the bound water associated with collagen, 4) the lipids in fat cells, and 5) the collagen and other biomacromolecules. Investigation of the cortical bones via ultrahigh‐field MR depends on the clear distinction between different pools of ^1^H signals. In principle, distinctions can be made between the spatial distribution of the signal intensity, the T_1_, T_2_, and T_2_* relaxation,^[^ ^20^, ^22^, ^24^ ^]^ the diffusivity,^[^ ^27^, ^28^, ^29^ ^]^ the chemical shift spectrum,^[^ ^12^, ^21^, ^22^, ^38^ ^]^ and the double‐quantum signal.^[^ ^28^ ^]^ Continuous efforts have been made to develop MR techniques and strategies for characterizing bones in vitro and in vivo.^[^ ^27^, ^39^, ^40^, ^41^ ^]^ However, previous studies based on relatively low magnetic fields (≤3 T) might not be transferrable to the data obtained under an ultrahigh field, because many MR properties are field‐dependent. Therefore, we performed comprehensive measurements on cortical bones under 14 T to identify the structural basis of different MR signatures.

### 2.1 UTE Imaging and Relaxation under 14 T

**Figure 2:**
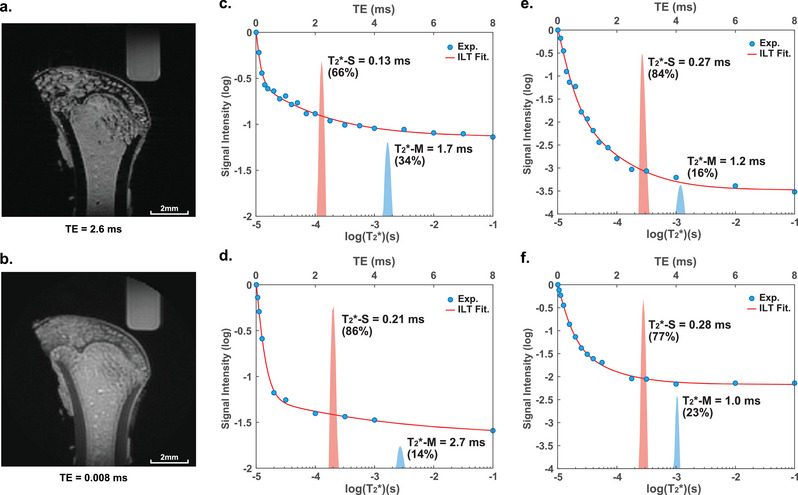
MRI and T_2_* distributions of cortical bones under 14 T. a) MSME image (TE = 2.6 ms) and b) UTE image (TE = 8 µs) of a rat femur. The UTE image shows visible signals at the cortical area, whereas MSME shows no cortical signal. The UTE T_2_* decay curves of c) rat, d) sheep, e) swine, f) bovine cortical bones. The blue dots are the experimental results; the red lines are the fitting curves of ILT. The ILT fittings show bi‐exponential decays with a short T_2_* (T_2_*‐S = 0.1–0.5 ms) and a medium T_2_* (T_2_*‐M = 1–4 ms).

The major characteristic of ^1^H signals in cortical bones under 14 T is their short T_2_/T_2_* relaxation time (at the order of 0.1–10 ms). The fast relaxation leads to an almost complete absence of the ^1^H signal of the cortical bone on typical MR images. For example, the voxels of the cortical bone in rat femurs appear completely dark on MR images obtained using multi‐slice‐multi‐echo (MSME) pulse sequences with an echo time (TE) of 2.6 ms (**Figure** **2**a). The special UTE sequence greatly enhances signals with short T_2_* by reducing the TE to as short as 8 µs. Images acquired with UTE show reasonable signal intensity in the cortical bone area (Figure 2b). Using the UTE sequence and selecting the cortical area (Figure S1, Supporting Information), we obtained the T_2_* (i.e., the effective T_2_)^[^ ^42^ ^]^ decay curves from freshly harvested cortical tissues on the long bones of rats, sheep, swine, and bovine (Figure 2c–f). The T_2_* curves exhibit multi‐exponential decay features indicating a distribution of T_2_* values. It is convenient to use inverse Laplace transform (ILT) to identify the distribution of T_2_* components. ILT is an algorithm that transforms the decaying signal into a sum of discrete principle components, each with a unique decay constant and amplitude.^[^ ^43^ ^]^ We further validate the goodness of least square fittings of multi‐exponential curves (Figure S2, Supporting Information). The results show the UTE decays are composed of a short T_2_* component of 0.1–0.5 ms (red peak) and a medium T_2_* component of 1–4 ms (blue peak).^[^ ^44^ ^]^ Compared with the results obtained on clinical MRI scanners of lower magnetic fields^[^ ^20^, ^24^, ^26^, ^30^, ^32^ ^]^ (Table S1, Supporting Information), the stronger magnetic susceptibility at 14 T reduces the values of T_2_*, but the multi‐exponential decay remains. The relative fractions of the fast and slow relaxing components vary in different bones, likely due to intrinsic species differences or to the variances in sample preparation (see Supporting Information). These T_2_* results show high reproducibility in bone samples, such as those of mice and rats (Figures S3 and S4, Supporting Information).

### 2.2 MR Spectroscopy of Cortical Bone at 14 T

**Figure 3:**
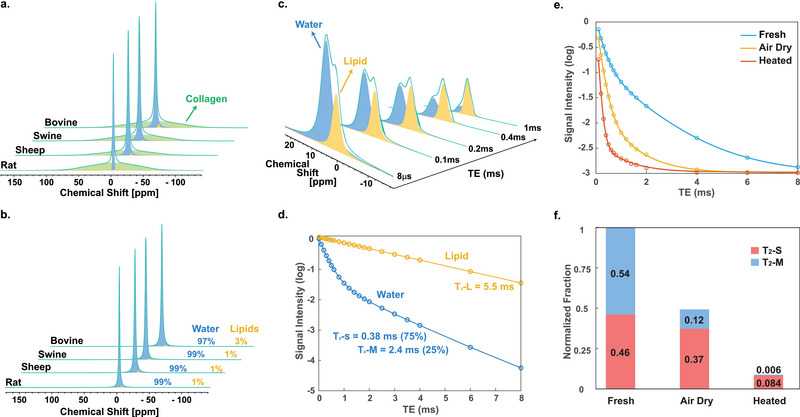
^1^H MRS of cortical bones under different sample treatments. a) Direct excitation spectra and b) spin‐echo spectra (TE = 8 µs) of cortical bones. Water and lipid fractions are shown next to each spectrum. c) A series of ^1^H spectra collected with the spin‐echo sequence with different echo times on an air‐dried swine bone. d) T_2_ decay curves of the water and lipids (according to respective ^1^H peaks on MRS) in the air‐dried swine bone. e) T_2_ relaxation curves of swine cortical bone under different sample treatments. f) Fractions of short and medium T_2_ components for the curves obtained in (e). The water content of each sample is referenced to a water phantom using voxel‐wise signal intensity in the UTE image (see Supporting Information).

To understand the origins of the different T_2_* components, we performed complementary MRS experiments at 14 T. In principle, the T_2_* obtained by UTE corresponds to the decay rate of free induction decay detected in MRS. The time‐domain decay rate is inversely related to the full‐width‐at‐half‐maximum (FWHM) of frequency‐domain peaks after Fourier transform: $\mathrm{FWHM}\;=1/(\pi \times T_{2}^{*})\;$. Additionally, the spectra in MRS can sort the ^1^H signals of different resonant frequencies. The ^1^H spectra of mammalian bones are composed of three peaks (**Figure** **3**a): the main signal at 5 ppm (corresponds to the chemical shift of water, colored in blue), a tiny peak at about 1 ppm (corresponds to the chemical shift of lipids, shown in yellow), and a broad pattern with an FWHM of ≈30 kHz (colored in green).

**Figure 4:**
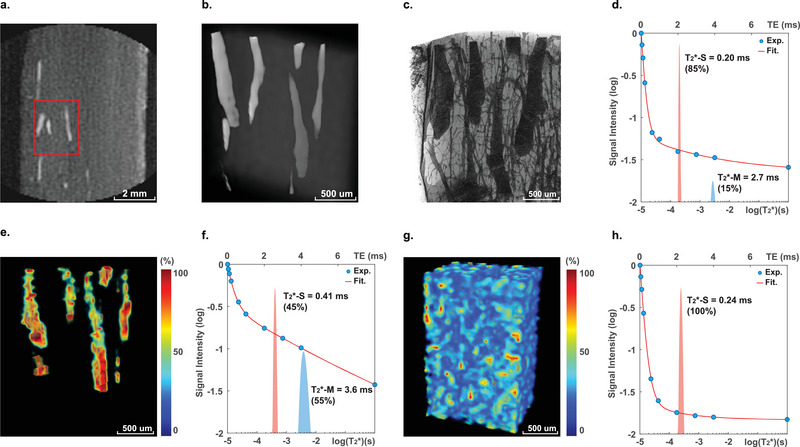
Ultrahigh‐resolution MRI of sheep cortical bone. a) A 2D cross‐section of a UTE image of cortical tissue from a sheep femur at a spatial resolution of 62.5 µm (field of view: 8×8×8 mm^3^, matrix size: 128×128×128). b) 3D UTE reconstruction of the ^1^H intensity of the selected cortical bone region. c) Micro‐CT image of the same region. The spatial resolution of the micro‐CT is 1 µm. d) T_2_ distribution of the mean signal in the whole volume of the UTE reconstruction. e) Color map of the T_2_*‐M fraction and f) T_2_* distribution in the regions of the Haversian canals. g) Color map of the T_2_*‐M fraction and h) T_2_* distribution in the regions of the collagen matrix.

We attribute the 30‐kHz broad pattern to the macromolecular collagens which have strong ^1^H‐ ^1^H dipolar coupling of their protons. The FWHM of 30 kHz corresponds to a T_2_* of <0.01 ms, which is beyond the detection range of UTE experiments. The collagen signal is filtered out with the spin‐echo sequence with an echo time of 8 µs (Figure 3b). Only water and lipid signals are detected after the spin‐echo filter. In the animal samples, the spectra are dominated by the water signal at 5 ppm. The water signal shows a bi‐exponential decay with a short T_2_ of ≈0.3 ms (T_2_‐S) and a medium T_2_ of 2–4 ms (T_2_‐M) (**Figure** **4**d and Figure S5, Supporting Information). This confirms that the water signal contributes to the bi‐exponential decay in the UTE experiments (Figure 2c–f). The T_2_ values measured by spin‐echo are slightly larger than the T_2_* values measured via UTE because the T_2_* also includes the effect of field inhomogeneity.^[^ ^42^ ^]^ In the fresh bone samples, the lipid signal intensity is quantitatively insignificant (<3%) compared with that of water (Figure 3a,b). However, partially removing the water by air drying enables visualizing the lipid signals (Figure 3c). The spin‐echo experiment reveals that the lipid signal in swine bone has a T_2_ of 5.5 ms (T_2_‐L) (Figure 3d).

### 2.3 Identification of Free and Bound Water

To further identify the structural basis of the water signal, we performed spin‐echo experiments on cortical bones after different sample treatments. The swine bones were prepared under three conditions: fresh, air‐dried for 12 h, and additional heating at 100 °C for 1 h (Figure 3e). For these samples, we deduce the total water content from the signal intensity (by referencing to a water phantom) and derive the relative T_2_ fractions from bi‐exponential fittings of the spin‐echo decaying curves (Figure 3f).

In the fresh bone, the short and medium T_2_ fractions are ≈50% each. In the air‐dried sample, the total water content is reduced by 50%. The removed water comes mostly from the free water in Haversian systems (with a T_2_‐M). In living or fresh tissue, the Haversian–Volkmann canals are filled with blood capillaries, nerve fibers, and lymphatic vessels, i.e., a high concentration of free‐flowing water.^[^ ^45^, ^46^ ^]^ The remaining T_2_‐M water content (12%) after air drying is attributed to the trapped water in the lacuno‐canalicular system, which hosts osteocytes and their interconnected dendrites.^[^ ^45^, ^46^ ^]^

More than 90% of the water is eliminated by the additional heating process. The T_2_‐S component that survived after heating should have come from collagen‐bound water because these bound water molecules are strongly held by hydroxyl‐rich collagens.^[^ ^29^, ^47^ ^]^ In short, the T_2_‐M component corresponded to the water in both the Haversian–Volkmann canals and the lacuno‐canalicular system, and the T_2_‐S component corresponded to the collagen‐bound water.

### 2.4 3D Reconstruction of Haversian Canals

With the relaxation characteristics identified, we used UTE imaging to map the microscopic anatomy in cortical bones, focusing on the Haversian canals. In small mammals such as rat and mouse, the Haversian canals have a diameter of ≈10 µm.^[^ ^48^ ^]^ In larger mammals, including sheep and human, the Haversian canal diameter reaches ≈100 µm.^[^ ^48^ ^]^ The Haversian system in cortical bones is typically observed in vitro with optical microscopy or micro‐CT reconstructions.^[^ ^49^, ^50^, ^51^ ^]^

Even with the UTE sequence, it is still difficult for a normal MRI scanner to resolve the canal structures in bone. The reported spatial resolution of UTE imaging is typically >200 µm^[^ ^24^, ^25^, ^26^, ^30^, ^32^ ^]^ (Table S2, Supporting Information). The spatial resolution of MRI is limited due to a combination of reasons such as the strength of the magnetic field, the water concentration in a voxel, and the gradient strength. However, the ultrahigh field of 14 T and the strong gradient of up to 1.4 T·m^−1^ enabled the micron‐scale resolution in the cortical bone up to 20 µm (Figures S6 and S7, Supporting Information).

Figure 4a shows a longitudinal section of sheep bone (a cortical bone slice extracted from a sheep femur) acquired via 3D UTE imaging. The best image quality was attained under a spatial resolution of 62.5 µm (field of view: 8×8×8 mm^3^, matrix size: 128×128×128, with a 0.7 T·m^−1^ gradient). This longitudinal section shows canal‐like structures with strong ^1^H intensity (the bright areas). 3D reconstruction of a selected region reveals water‐filled canals with diameters of 100–200 µm, which are attributed to the Haversian canals in the cortical bone (Figure 4b). To validate the structure identification, we obtained micro‐CT imaging of the same sample with a resolution of 1 µm (Figure 4c). The micro‐CT provides an inverse image of the MRI as it probes the mineral matrix. Several dark tubes (i.e., the Haversian canals) are visible on the micro‐CT image, which exactly match the position and sizes of the UTE‐detected canals. The thinner dendritic structures with diameters of 10–50 µm (i.e., the canalicular network) are too small to be resolved in the UTE images. In Table S3 (Supporting Information), we show the bones with increased porosity (as determined by micro‐CT) also have an increased fraction of pore water (i.e., the fraction of T_2_*‐M, as determined by MRI). The result confirms the consistency between the two different imaging methods.

In addition to the ^1^H signal intensity map, UTE also provides a map of spatially resolved T_2_* relaxivity. The short and medium T_2_* fractions can be deconvoluted for different cortical regions. The whole sample volume processes 85% of the short T_2_* (T_2_*‐S) and 15% of the medium T_2_* (T_2_*‐M) (Figure 4d). But for the Haversian canals (Figure 4e), the fraction of the T_2_*‐M reaches 60%, corresponding to free pore water (Figure 4f). The remaining 40% of the T_2_*‐S fraction comes from the interfacial regions where the voxels of the collagen matrix and the canals are inseparable. In the collagen matrix (Figure 4g), the ^1^H signal is dominated by the T_2_*‐S showing mostly collagen‐bound water (Figure 4h). Therefore, the fraction maps of T_2_* relaxivity can be classified as the Harversian canals (Figure 4e) and collagen matrix (Figure 4g).

### 2.5 Spatial Distributions of Water and Lipids

**Figure 5:**
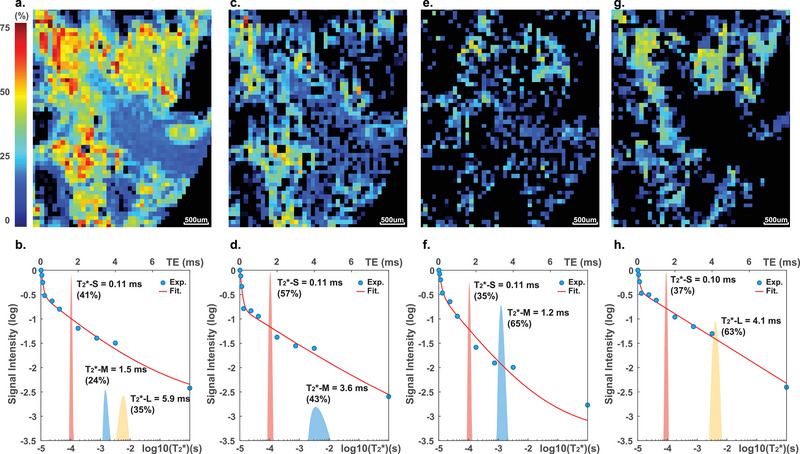
Ultrahigh‐resolution MRI of human pericortical tissue with osteoarthritis (OA‐1). a) 2D ^1^H intensity color map from a UTE image at a resolution of 62.5 µm (field of view: 8×8×20 mm^3^, matrix size: 128×128×128). b) T_2_* distribution of the whole cross‐section. c) Color map of the T_2_*‐S fraction and d) T_2_* distribution of the same region. The T_2_*‐S region represents the collagen matrix compartment. e) Color map of the T_2_*‐M fraction and f) T_2_* distribution of the same region. The T_2_*‐M region represents the free pore water compartment. g) Color map of the T_2_*‐L fraction and h) T_2_* distribution of the same region. The T_2_*‐L region represents the lipid compartment.

In addition to the animal samples, we studied the relaxation and UTE images of human specimens of pericortical tissues near the hip joint. The specimens were extracted from the tissues excised during joint replacement surgeries for patients with long‐term osteoarthritis (OA) or osteoporosis (OP). The samples were obtained from the pericortical region of the femoral neck, where the cortical and trabecular bone merge (Figure S8, Supporting Information). **Figure** **5**a shows the ^1^H signal intensity map in a representative cross‐section under a spatial resolution of 62.5 µm. The pores are wider in the pericortical region,^[^ ^52^, ^53^ ^]^ and the water inside can easily be lost during sample preparation, resulting in voids of ^1^H signals (dark spots).

Human pericortical specimens show a much higher lipid content than that of animal bones (Figure S8, Supporting Information). The high lipid content is attributed to the trabecular feature of pericortical bone and to the patients’ disease progression.^[^ ^54^, ^55^ ^]^ In the micron‐resolution UTE images, the T_2_* decay of the specimen (Figure 5b) also shows a triexponential curve consisting of T_2_*‐S (0.1–0.5 ms), T_2_*‐M (1–4 ms) and T_2_*‐L (4–8 ms).

We further plot the fraction maps of the three ^1^H relaxivities (Figure 5c,e–g) and the T_2_* distributions of each compartment (Figure 5d,f,h). These maps provide the classifications of the collagen matrix (T_2_*‐S), as well as water (T_2_*‐M) and lipids (T_2_*‐L) in trabecular or pathological pores (the lipids are mostly included in adipocytes^[^ ^56^ ^]^). The colors represent the relative fractions of each compartment in an individual pixel. The classification maps separate the areas of the collagen matrix (Figure 5c), pore structures (Figure 5e), and lipids (Figure 5g). Again, there are more than a single relaxation component in each map because of the structural overlaps in the voxels.

Similar plots are provided for the pericortical bone specimens of different patients (Figures S9–S11, Supporting Information). We performed comprehensive analysis of the voxel densities for the T_2_* fittings of UTE decay curves (Figures S12‐S15, Supporting Information). The statistical results show that the pericortical tissues with OA have higher lipid content than those with OP (Figure S16, Supporting Information), which is consistent with the previous study.^[^ ^57^ ^]^ It suggests that the volumetric density and distributions of water/lipid compartments may be pathological indications of bone tissues.

## 3 Discussion

A few imaging tools allow investigation of the bone matrix at the micron level. Currently, micro‐CT is the preferred non‐destructive technique to reveal the 3D porous structure of cortical tissue.^[^ ^58^, ^59^ ^]^ Otherwise, the specimen must be carefully sectioned and chemically processed to fit an optical or electron microscope for microscopic analysis.^[^ ^48^, ^50^ ^]^ These established techniques focus on the static structure of the mineralized matrix. Water and other molecular metabolites, which comprise ≈20% of the cortical volume, are not recorded in the structural images.

Conversely, MR signals are sensitive to the chemical and dynamic properties of water and molecular species arising from the different contrast mechanisms such as signal intensity, chemical shift, relaxation, and diffusion. Evolving MR techniques can provide complementary information on the physiological state of bone tissue and can show pathological developments.^[^ ^60^, ^61^, ^62^ ^]^ However, MRI on clinical scanners has insufficient spatial resolution to assess the localized histology.

Ultrahigh magnetic fields (e.g., 14 T) can increase the spatial resolution of MRI by approximately one order of magnitude. Micron‐level spatial resolution provides clear images of porous structures that are inaccessible on lower‐field scanners. The comparisons of MRI resolutions under different magnetic fields are shown in Table S2 (Supporting Information).

More importantly, the T_2_/T_2_* relaxation characteristics show details of the soft and organic compartments that are invisible in micro‐CT images. Note that the T_2_* values are field dependent, a stronger magnetic field usually leads to smaller T_2_* values (Table S1, Supporting Information). Besides measuring T_2_ relaxation, UTE‐MRI can be combined with various pulse sequences^[^ ^63^, ^64^, ^65^ ^]^ to extract comprehensive information about the local environment within the tissue. Micron‐resolution MRI offers great potential for pathological studies on ex vivo tissues with bone diseases or with lesions of tumor metastasis.^[^ ^66^, ^67^ ^]^ For instance, the 14 T MRI has been utilized to study structural changes in the hippocampus of human brains with Alzheimer's disease.^[^ ^68^ ^]^

On the other hand, there are several limitations of ultrahigh field MRI. First, ultrahigh magnetic field MRI (>7 T) has not been approved in clinical use. The potential safety concerns for a strong magnetic field are more critical than those for a lower magnetic field.^[^ ^69^ ^]^ Nevertheless, ultrahigh field MRI is currently under evaluation in frontier biomedical studies, e.g. the research of brain.^[^ ^70^, ^71^, ^72^ ^]^ It is possible for ultrahigh field MRI to be adapted gradually in future years. Second, the variations of tissue susceptibility may lead to a greater field inhomogeneity under a strong magnetic field, which poses problems for image and data processing. We tried to alleviate this issue by using a relatively small coil of 10 mm in diameter. In the future, various strategies may be used to improve the high‐field imaging of larger samples, including better radio‐frequency and gradient coil designs,^[^ ^73^, ^74^ ^]^ improved pulse sequences,^[^ ^73^, ^75^ ^]^ and advanced image processing algorithms.^[^ ^76^, ^77^ ^]^

In summary, we show that UTE imaging at 14 T can resolve the micron‐level anatomy of porosity, and UTE‐derived T_2_* relaxation allows classifying the spatial distributions of the collagen matrix, lipids, and pore water. We reveal the three‐dimensional anatomy of the Haversian canals in sheep femurs with an MRI resolution of 20–80 µm. We also uncover the separable spatial distributions of water and lipids in human bone specimens at a micron‐level resolution. The ability of 14 T MR to resolve cannular structures and distinguish soft compartments in cortical bones unlocks new potential of ultrahigh‐field MR for bone research both in vitro and in vivo.

## 4 Experimental Section

### Sources of Bone Samples

Cortical tissues from swine, bovine, and sheep were extracted from femurs bought fresh from a market and cut into suitable sizes for MR experiments. Wild‐type mice and rats were dissected to obtain complete femurs, and the muscle was removed. Human cortical tissue was collected from human patients who underwent joint replacement surgery. The patients were diagnosed with late‐stage OA or OP. The experimental procedures used in this study were approved by the ethics committee and institutional review board of the Ninth People's Hospital of Shanghai Jiao Tong University (approval number: SH9H‐20190T190‐2). Informed consents on enrollment had been signed and provided by all participants.

### Sample Treatments

Sample Cutting: Fresh samples were washed with deionized water to remove the surface fat and blood. The muscle and tissue outside the bone were removed with scissors and a scalpel, and the marrow inside the bone was removed with tweezers. For bigger mammals, the cortical bone was first cut to the approximate size using a chainsaw, then refined with an electric/manual grinder. Before the experiments, the prepared specimens were stored in a refrigerator at 4 °C in a phosphate‐buffered saline (PBS) solution.

Drying Treatment: A piece of swine cortical bone was put in a petri dish on the lab table for 12 h at room temperature (25 °C). After air‐drying, the cortical tissue was heated at 100 °C for 1 h in an air‐tight oven.

### Micro‐CT Sample Preparation

The same specimen of sheep bone was used for both MR and micro‐CT imaging and it allowed a direct comparison between images. After completing the MR experiments, the specimen was removed from the Fomblin (Solvay) and immersed in PBS for more than 3 days to remove the remaining Fomblin. The cortical tissue was then cut into 2.5×2.5×2.5 mm^3^ for micro‐CT experiments. Before micro‐CT scanning, the sample surface was wiped to remove the water.

### MR Experiments—Sample Preparation for MRS

Bone specimens were cut into small pieces to fit in a 5‐mm NMR tube. Fomblin was added to cover the samples, and the air was removed by vacuum. The NMR tubes were sealed with Parafilm and aligned perpendicular to the vertical magnetic field.

### Sample Preparation for MRI

For the bone samples from large mammals (i.e., swine, bovine, sheep, and human), a suitably sized section was cut from the middle of the cortical bone and fixed in a 10‐mm glass tube for MRI experiments (Figure S17, Supporting Information). For the mice and rats, the whole femur (with muscle partially removed) was placed in a 10‐mm glass tube for MRI experiments (Figure S17a, Supporting Information). The samples were immersed in Fomblin, and the tubes were slowly vacuumed to remove the air. After vacuuming, a phantom of deionized water was placed near the sample as a reference for calibrating the voxel‐wise ^1^H signal intensity. The tubes were sealed with Parafilm once the preparation was completed.

### Experimental Details of MR Measurements

MRS experiments were performed on a 9.4 T Bruker Avance III HD NMR instrument using a 5‐mm probe. The recycle delays and pulse length were optimized for each set of experiments. Direct excitation and spin echo sequences were used to obtain ^1^H spectra of the cortical bones.

MRI experiments were performed on the 14.1 T Bruker Avance III HD NMR instrument with a Bruker Micro2.5 micro‐imaging system. A 10‐mm volume coil was used. Table S4 (Supporting Information) lists the parameters used in the 3D‐UTE and MSME pulse sequences. Water phantoms were used to calibrate the signal intensity at variable echo times. The same UTE trajectory file was used for image reconstruction in each echo time to reduce artifacts. The images were obtained from Bruker Paravision 6.0 package. Additional image processing and visualization were performed on the MIPAV package and MATLAB^®^. All experiments were performed at the room temperature. Before each experiment, the radio‐frequency pulse power and the repetition time were carefully set to maintain a low duty cycle (<20%) to avoid sample heating.

### Processing of Relaxation Data

ILT was used for the initial evaluation of a new dataset. Tikhonov regularization term of ILT was added to avoid the bias of noise for the signals at long TE's.^[^ ^78^ ^]^ When ILT of the decay showed two or three T_2_/T_2_* components, the bi‐exponential or tri‐exponential fittings were used to compare the results. The Akaike information criterion (AIC)^[^ ^79^ ^]^ was used to evaluate the fitting goodness of the different methods (including ILT, single‐component, bi‐component, tri‐component). The least‐square fitting may be used if it yields the best‐fitting goodness in AIC model (see the examples in Figure S2, Supporting Information). In general, the results of ILT and least‐square fittings are similar.

## Conflict of Interest

The authors declare no conflict of interest.

## Supporting information

Supporting Information

## Data Availability

Research data are not shared.
