# Supplementary material for: Micron‐resolution Imaging of Cortical Bone under 14 T Ultrahigh Magnetic Field
Source: Adv Sci (Weinh). 2023 Jun 20;10(24):2300959. doi: 10.1002/advs.202300959 (PMC10460861; doi:10.1002/advs.202300959)
Supplement: Supplementary file 1 — Supporting Information [file ADVS-10-2300959-s001.pdf]

ADVANCED SCIENCE
Open Access

# Supporting Information

for *Adv. Sci.*, DOI 10.1002/advs.202300959

Micron-resolution Imaging of Cortical Bone under 14 T Ultrahigh Magnetic Field

*Tian He, Zhenfeng Pang, Yu Yin, Huadong Xue, Yichuan Pang, Haixin Song, Jianhua Li, Ruiliang Bai, An Qin and Xueqian Kong**

**Supplementary Information for**

# Micron-resolution Imaging of Cortical Bone under 14 T Ultrahigh Magnetic Field

Tian He[1], Zhenfeng Pang[1], Yu Yin[1], Huadong Xue[1,2], Yichuan Pang[3], Haixin Song[2], Jianhua Li[2], Ruiliang Bai[4,5], An Qin[3], Xueqian Kong[1,2]*

1. Department of Chemistry, Zhejiang University, Hangzhou 310027, China
2. Department of Rehabilitation, Sir Run Run Shaw Hospital, College of Medicine, Zhejiang University, Hangzhou 310016, China
3. Shanghai Key Laboratory of Orthopedic Implants, Department of Orthopaedics, Shanghai Ninth People's Hospital, Shanghai Jiao Tong University School of Medicine, Shanghai, 200011, China.
4. Interdisciplinary Institute of Neuroscience and Technology (ZIINT), College of Biomedical Engineering and Instrument Science, Zhejiang University, Hangzhou, China
5. School of Medicine, Zhejiang University, Hangzhou, China
6. Institute of Translational Medicine, Shanghai Jiaotong University, Shanghai, 200011, China

* e-mail: kxq@zju.edu.cn

**This PDF file includes:**

Data Processing Methods
Figures S1 to S17
Tables S1 to S4

# Data Processing Methods

## Image processing for MRI

Image processing and relaxation analyses were performed in MATLAB®. The MIPAV package was used for the 3D visualization of the UTE-MRI images. For $T_2$* relaxation analysis on the UTE-MRI, sections containing the cortical bone were selected. The region of interest (ROI) of the cortical tissue was determined on each slice (red ROI in Fig. S1b).

The mean signal of the water phantom (blue ROI) in each TE was used to calibrate the signal intensity of the ROI. For each voxel in the ROI, ILT or multi-exponential fittings were used to fit the $T_2$* decay curves. Table S4 lists the fitting parameters.

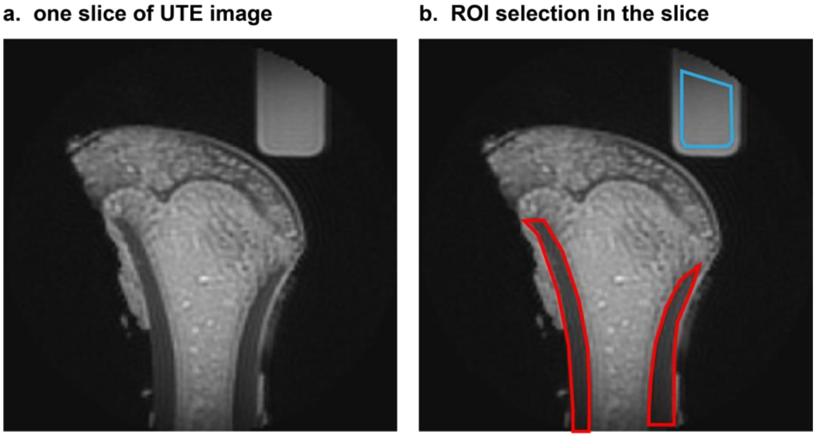


**Fig. S1 | ROI selection examples.** (**a**) original slice image on UTE-MRI. (**b**) ROI selection of water (blue) and cortical bone (red) for this slice.

## Fitting methods for relaxation data

Typically, ILT was first used to find the position and distribution of $T_2$* components for new datasets. ILT included a range of $T_2$/$T_2$* components from $10^{-5}$ to $10^{0}$ s and with 100 data points on a logarithmic scale. Single-exponential, bi-exponential, and tri-exponential fittings were also carried out by nonlinear least squares curve-fitting with the Levenberg-Marquardt algorithm and were tested under the Akaike information criterion (AIC)[66]:

$$\mathrm{AIC} = 2k - 2\ln(L)\,,\#(1)$$

where $k$ is the number of independently adjusted parameters within the model, and $L$ is the maximum likelihood. When the errors in the model follow an independent normal distribution, and when $n$ is the number of observations, and the sum square of residue (SSR) is the residual

sum of squares, the AIC becomes:

$$AIC = 2k + n\ln\left(\frac{SSR}{n}\right) \#(2)$$

AIC measures the goodness of the data fitting. The most appropriate fitting corresponds to the fitting with the smallest AIC value. For the data of sheep bone in Fig. S2, the bi-exponential fitting yields the smallest AIC value. For the data of human bone in Fig. S2, the tri-exponential fitting yields the smallest AIC value.

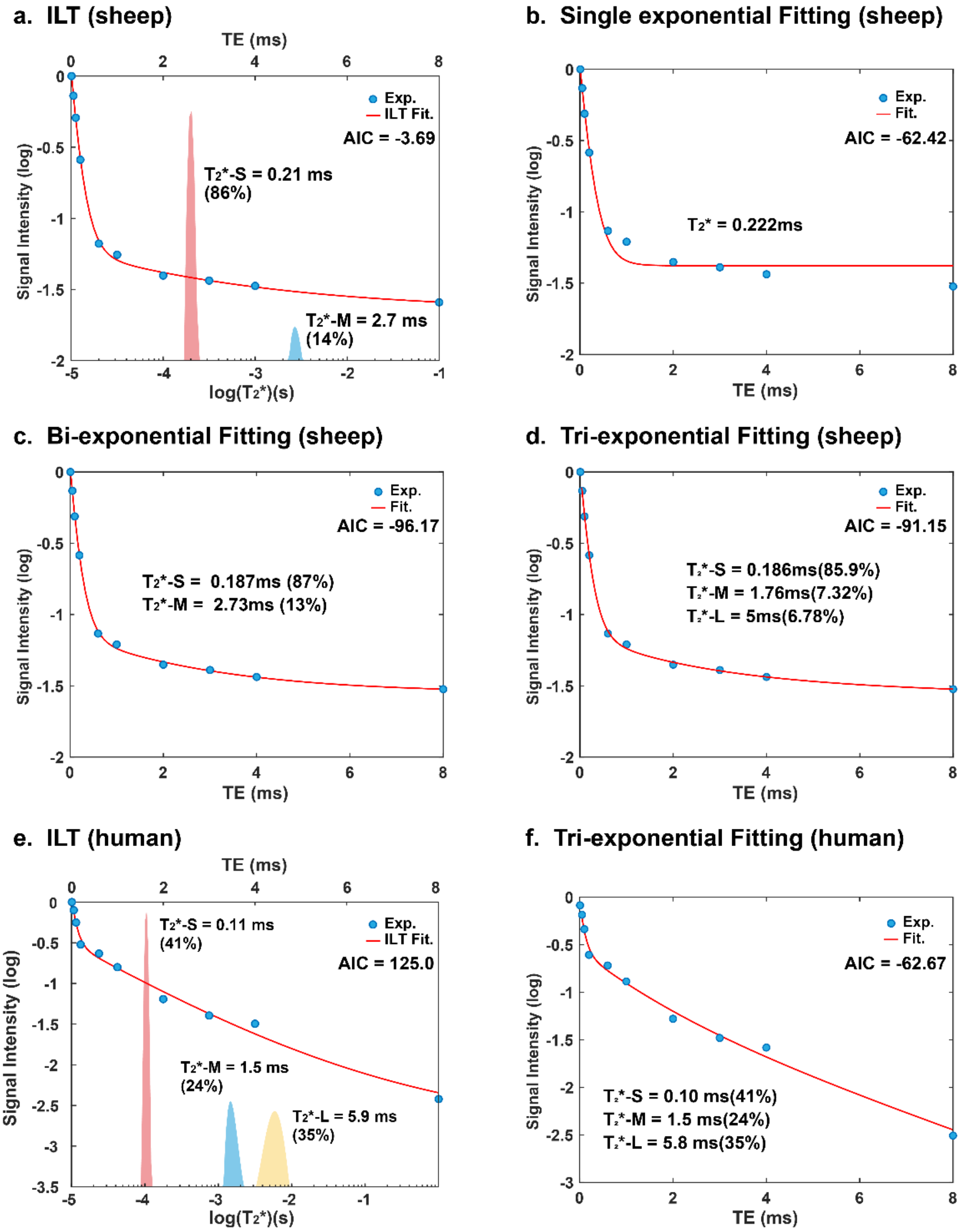


**Fig. S2** | (**a**) ILT, (**b**) single-exponential fitting, (**c**) bi-exponential fitting, and (**d**) tri-exponential fitting of T2* decay for sheep cortical bone. (**e**) ILT and (**f**) tri-exponential fitting of T2* decay for human bone.

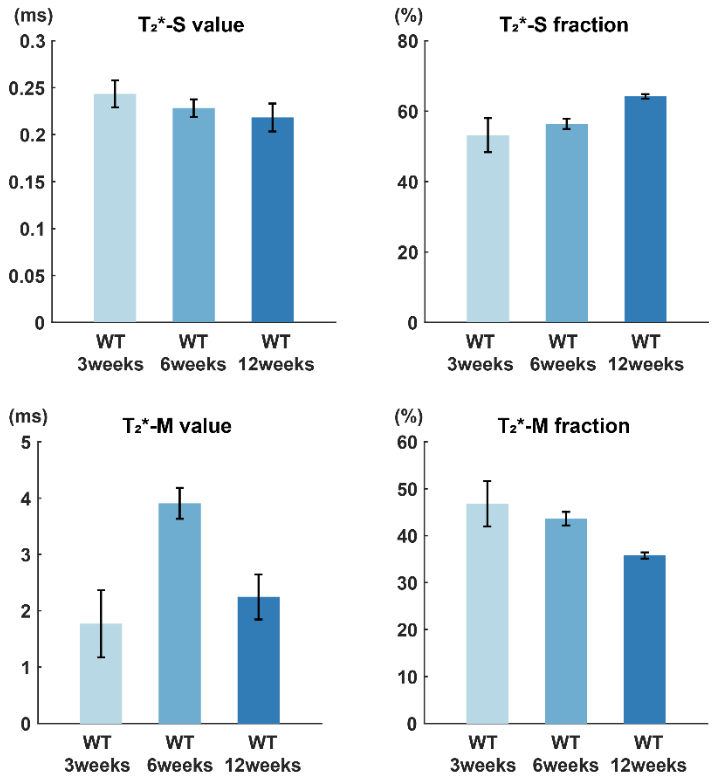


**Fig. S3 |** The $T_2$* fitting results for the cortical femur bone of wild-type mice of 3, 6 and 12 weeks. The error bar represent the standard deviation of multiple bone samples.

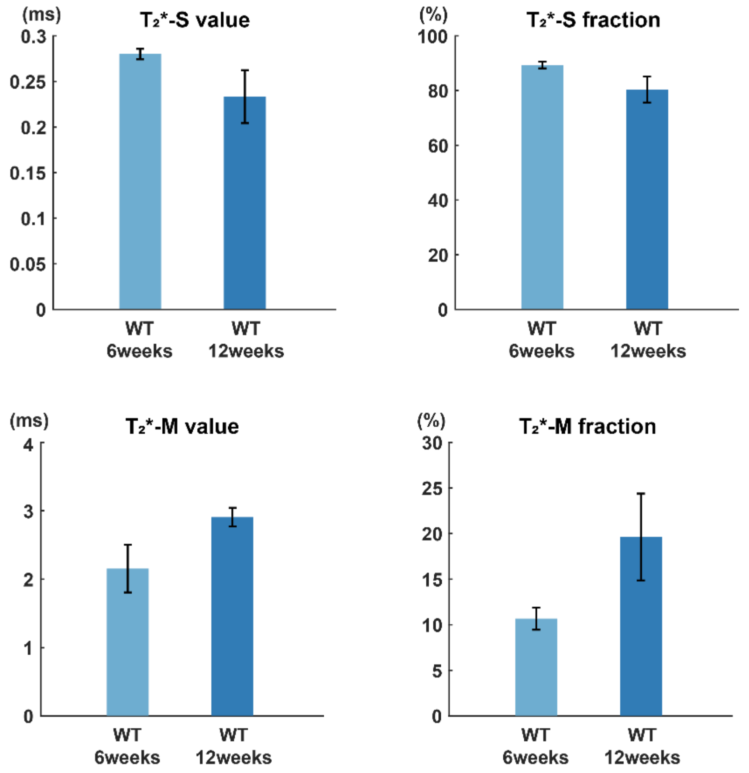


**Fig. S4 |** The $T_2$* fitting results for the cortical femur bone of wild-type rats of 6 and 12 weeks. The error bar represent the standard deviation of multiple bone samples.

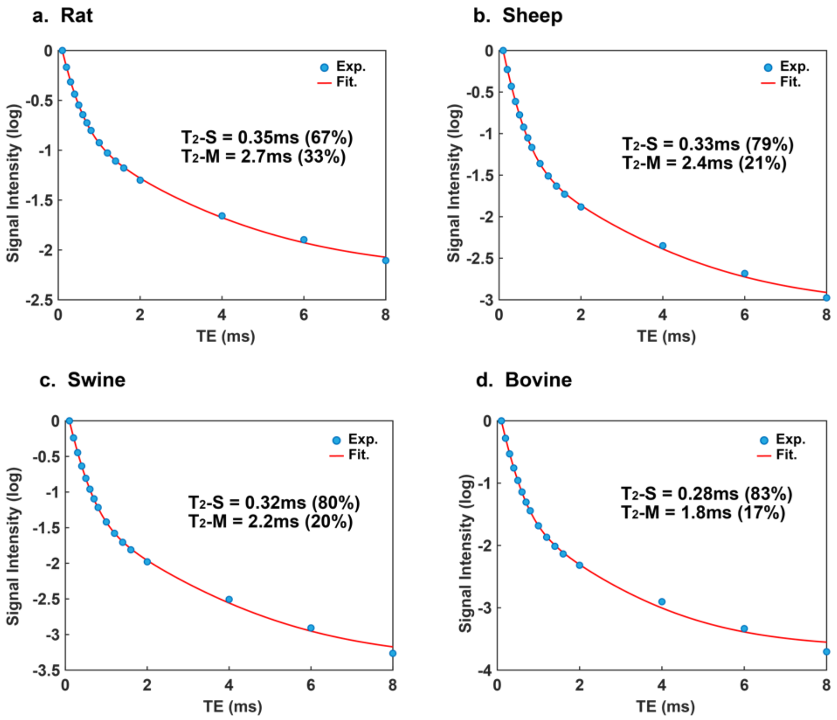


**Fig. S5 |** Bi-exponential fittings of the spin-echo decays of the water signal of cortical bones studied in the MRS experiments. (**a**) rat, (**b**) sheep, (**c**) swine, and (**d**) bovine.

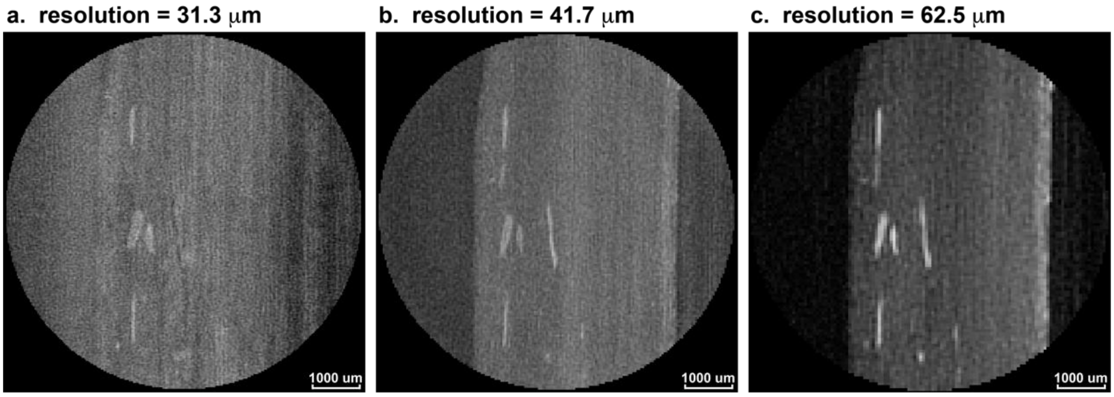


**Fig. S6 |** UTE images of a sheep cortical bone at resolutions of (**a**) 31.3 μm (FOV: 8×8×8 $mm^3$, matrix size: 256×256×256), (**b**) 41.7 μm (FOV: 8×8×8 $mm^3$, matrix size: 192×192×192), and (**c**) 62.5 μm (FOV: 8×8×8 $mm^3$, matrix size: 128×128×128).

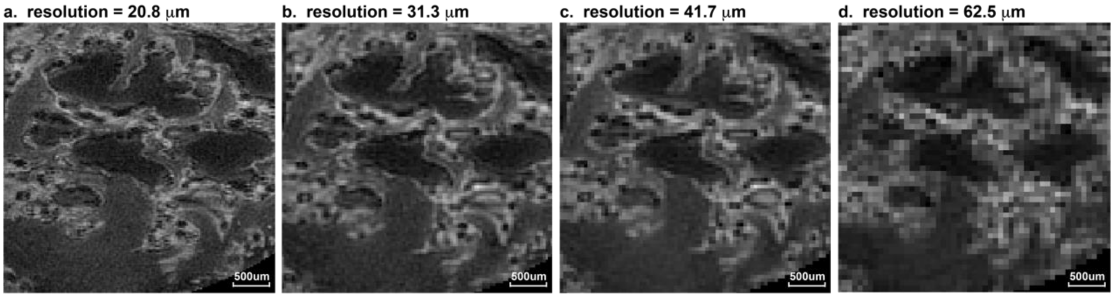


**Fig. S7 |** UTE images of a human cortical bone at resolutions of (**a**) 20.8 μm (FOV: 8×8×20 $mm^3$, matrix size: 384×384×384), (**b**) 31.3 μm (FOV: 8×8×20 $mm^3$, matrix size: 256×256×256), and (**c**) 41.7 μm (FOV: 8×8×20 $mm^3$, matrix size: 192×192×192), and (**d**) 62.5 μm (FOV: 8×8×20 $mm^3$, matrix size: 128×128×128).

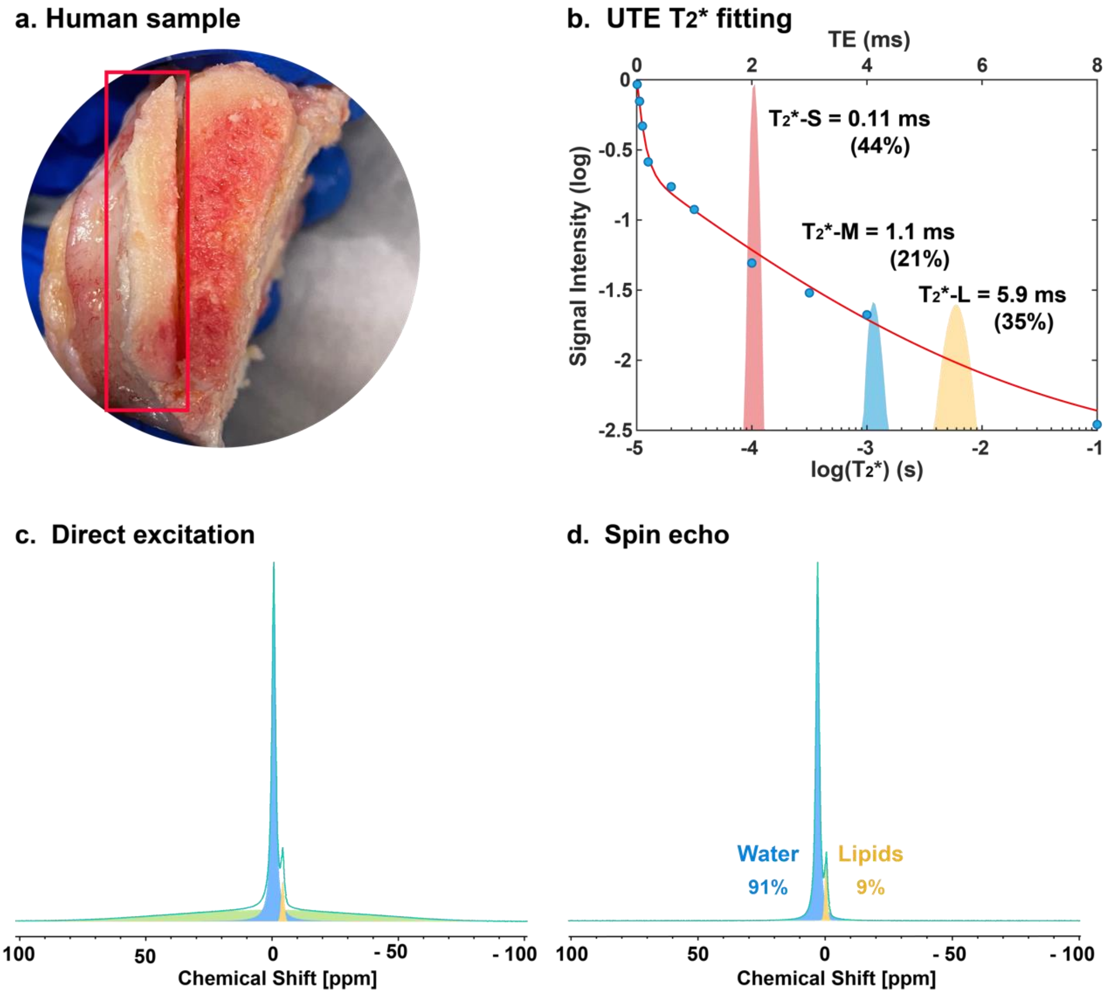


**Fig. S8** | (**a**) Excised tissue from the femoral neck of an 80-year-old patient who underwent joint replacement surgery. The red box indicates the extracted specimen for the MR experiments. (**b**) UTE $T_2$* decay of the human specimen at 14 T and the ILT fitting. (**c**) Direct excitation spectrum and (**d**) spin-echo spectrum (TE = 8 μs) of the human specimen. According to the deconvolution of the $^{1}$H spectra, the overall lipid fraction of the human specimen was 9%.

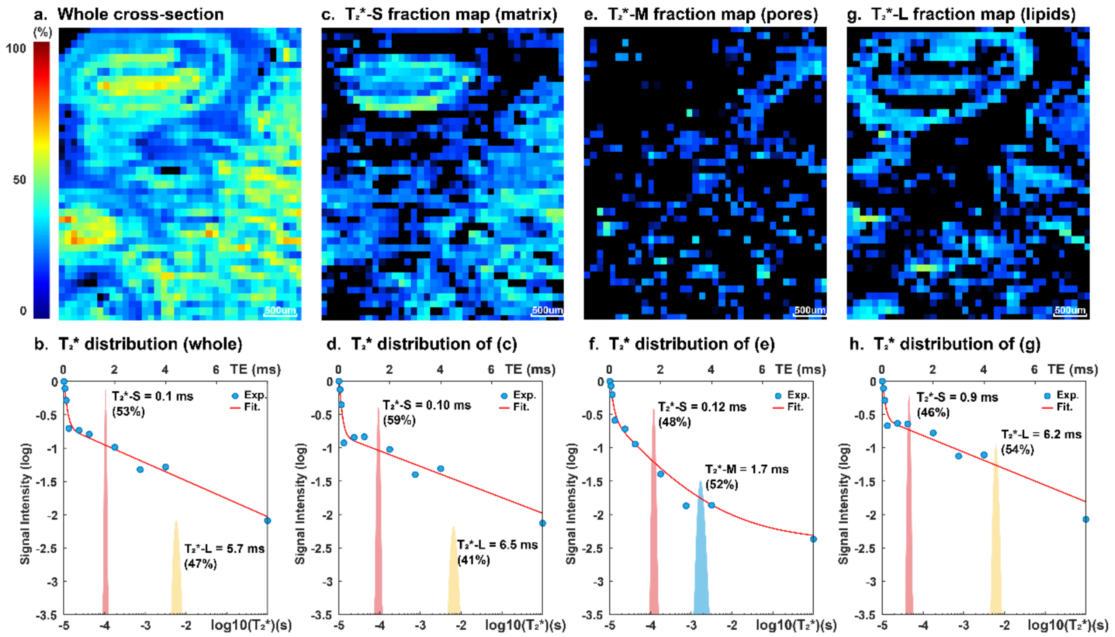


**Fig. S9 | Ultrahigh-resolution MRI of human peri-cortical tissue with osteoarthritis (OA-2).** (**a**) 2D $^1$H intensity color map obtained from a UTE image at a resolution of 62.5 μm (FOV: 8×8×20 mm$^3$, matrix size: 128×128×128). (**b**) $T_2$* distribution of the whole cross-section. (**c**) Color map of the $T_2$*-S fraction and (**d**) $T_2$* distribution of the same region. (**e**) Color map of the $T_2$*-M fraction and (**f**) $T_2$* distribution of the same region. (**g**) Color map of the $T_2$*-L fraction and (**h**) $T_2$* distribution of the same region.

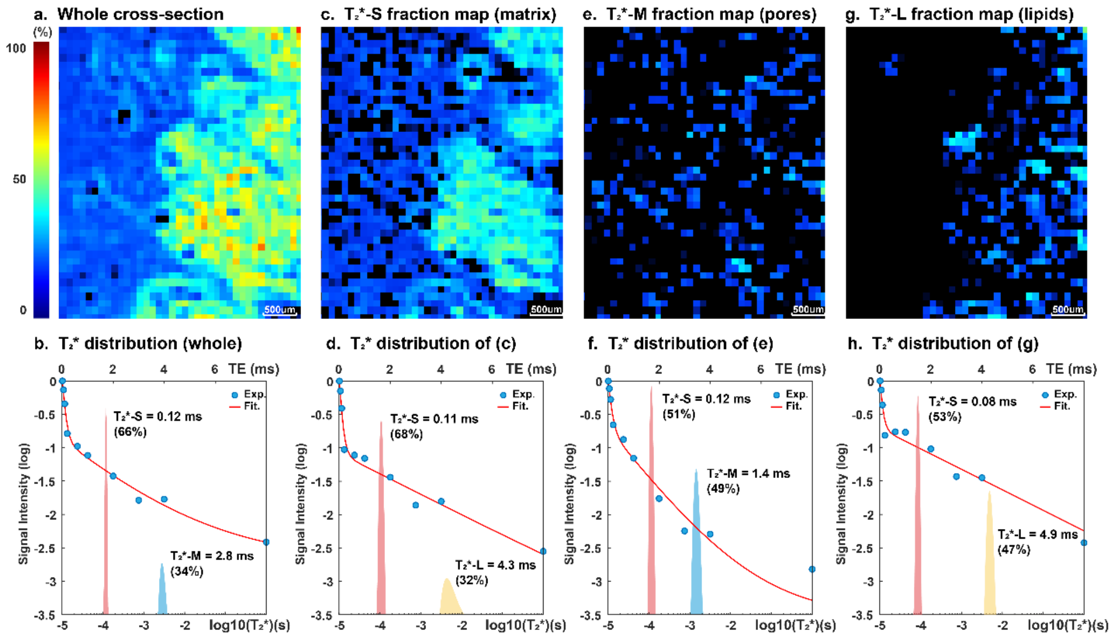


**Fig. S10 | Ultrahigh-resolution MRI of human peri-cortical tissue with osteoporosis (OP-1).** (**a**) 2D $^{1}$H intensity color map obtained from a UTE image at a resolution of 62.5 μm (FOV: 8×8×20 mm$^3$, matrix size: 128×128×128). (**b**) $T_2$* distribution of the whole cross-section. (**c**) Color map of the $T_2$*-S fraction and (**d**) $T_2$* distribution of the same region. (**e**) Color map of the $T_2$*-M fraction and (**f**) $T_2$* distribution of the same region. (**g**) Color map of the $T_2$*-L fraction and (**h**) $T_2$* distribution of the same region.

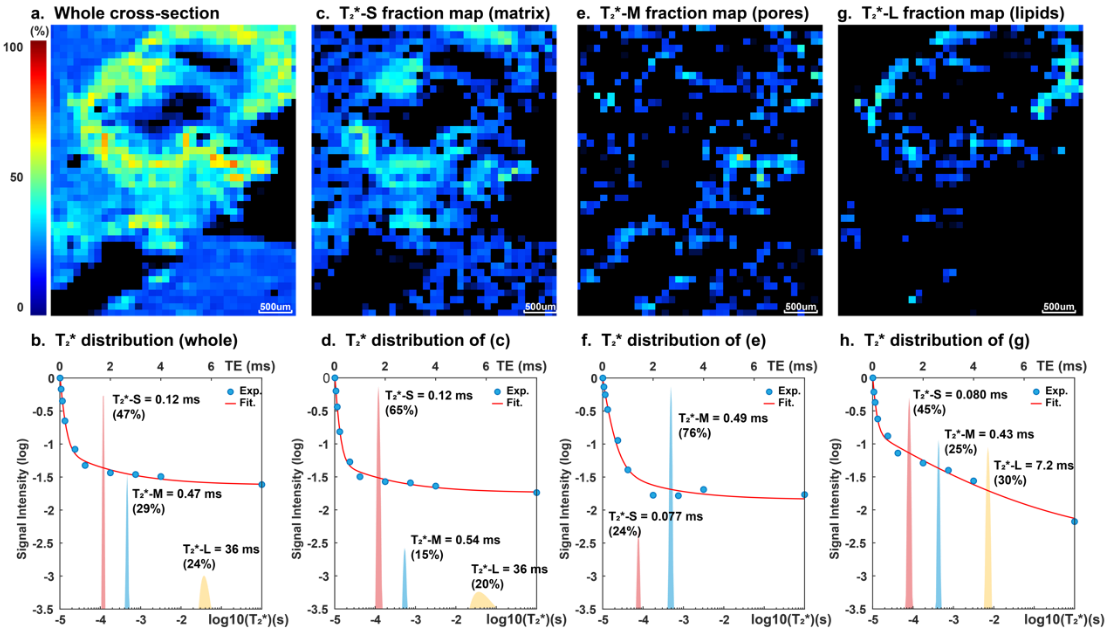


**Fig. S11 | Ultrahigh-resolution MRI of human peri-cortical tissue with osteoporosis (OP-2).** (**a**) 2D $^{1}$H intensity color map obtained from a UTE image at a resolution of 62.5 μm (FOV: 8×8×20 mm$^3$, matrix size: 128×128×128). (**b**) $T_2$* distribution of the whole cross-section. (**c**) Color map of the $T_2$*-S fraction and (**d**) $T_2$* distribution of the same region. (**e**) Color map of the $T_2$*-M fraction and (**f**) $T_2$* distribution of the same region. (**g**) Color map of the $T_2$*-L fraction and (**h**) $T_2$* distribution of the same region.

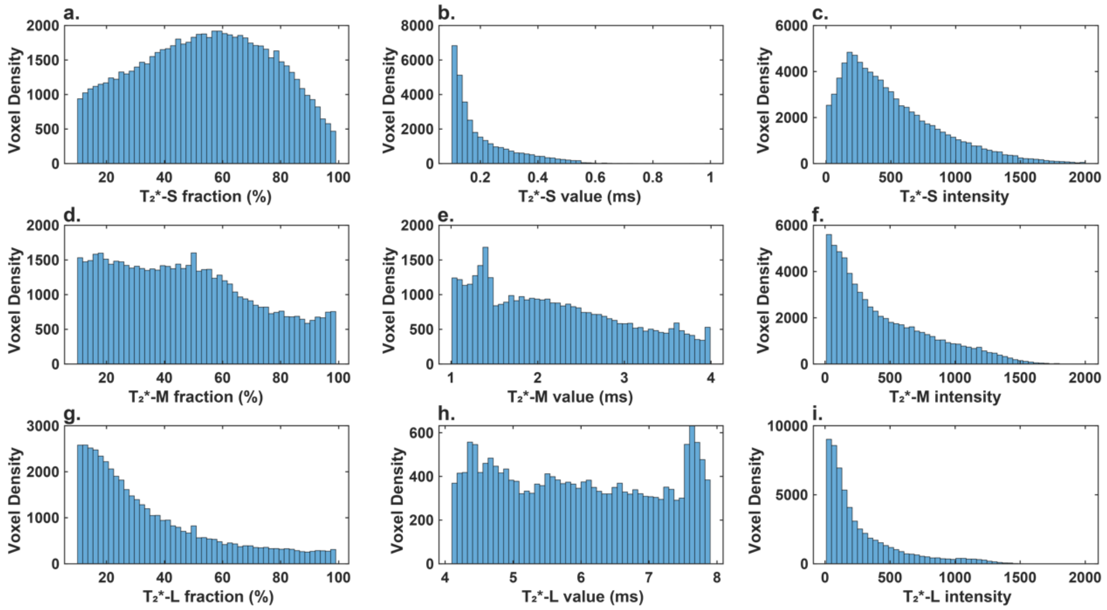


**Fig. S12 | Voxel density histograms of $T_2$* fittings on a human peri-cortical tissue with osteoarthritis (OA-1).** (**a**) $T_2$*-S fraction, (**b**) $T_2$*-S value, (**c**) $T_2$*-S intensity, (**d**) $T_2$*-M fraction, (**e**) $T_2$*-M value, (**f**) $T_2$*-M intensity, (**g**) $T_2$*-L fraction, (**h**) $T_2$*-L value and (**i**) $T_2$*-L intensity.

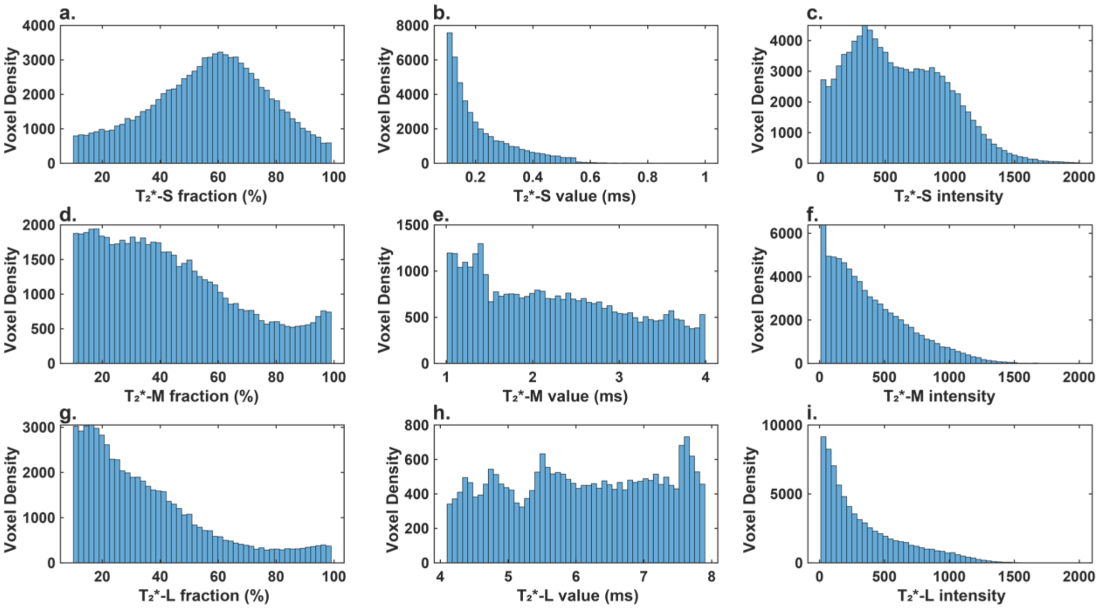


**Fig. S13 | Voxel density histograms of $T_2$* fittings on a human peri-cortical tissue with osteoarthritis (OA-2).** (**a**) $T_2$*-S fraction, (**b**) $T_2$*-S value, (**c**) $T_2$*-S intensity, (**d**) $T_2$*-M fraction, (**e**) $T_2$*-M value, (**f**) $T_2$*-M intensity, (**g**) $T_2$*-L fraction, (**h**) $T_2$*-L value and (**i**) $T_2$*-L intensity.

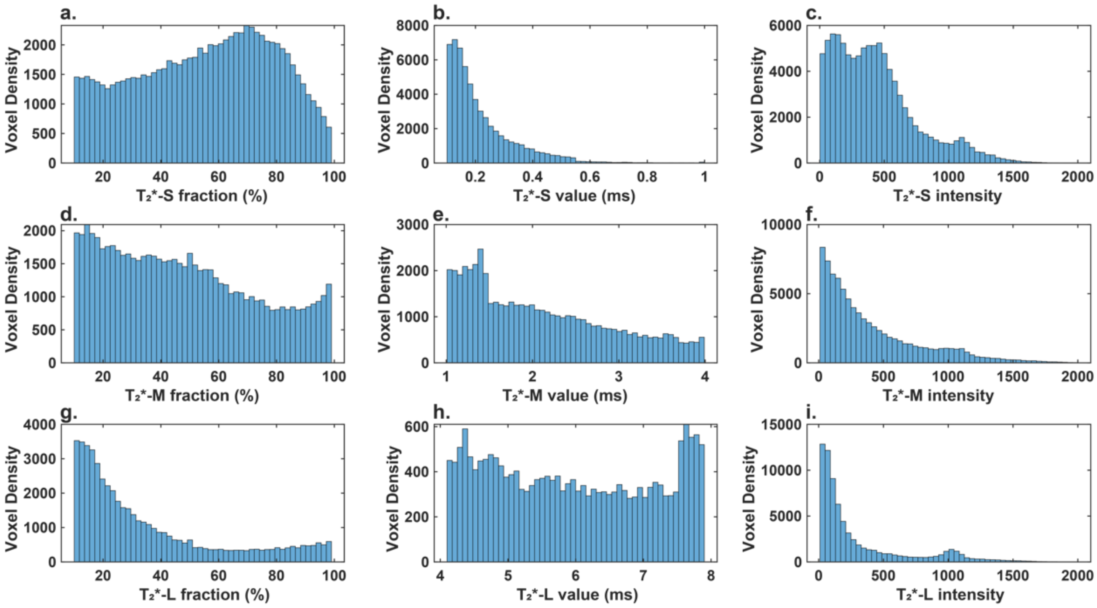


**Fig. S14 | Voxel density histograms of $T_2$* fittings on a human peri-cortical tissue with osteoporosis (OP-1).** (**a**) $T_2$*-S fraction, (**b**) $T_2$*-S value, (**c**) $T_2$*-S intensity, (**d**) $T_2$*-M fraction, (**e**) $T_2$*-M value, (**f**) $T_2$*-M intensity, (**g**) $T_2$*-L fraction, (**h**) $T_2$*-L value and (**i**) $T_2$*-L intensity.

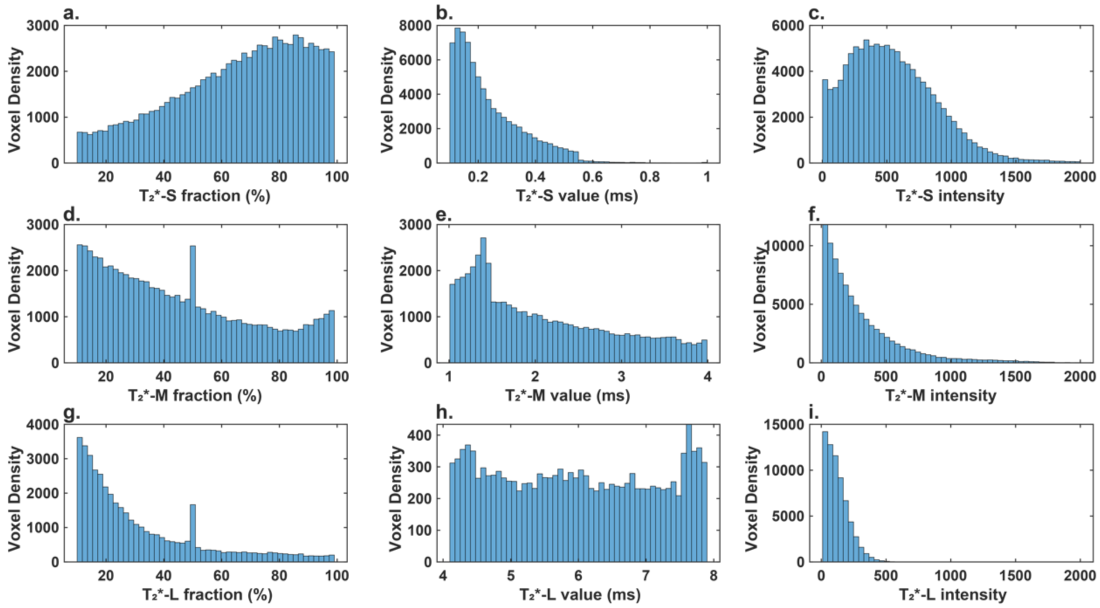


**Fig. S15 | Voxel density histograms of $T_2$* fittings on a human peri-cortical tissue with osteoporosis (OP-2).** (**a**) $T_2$*-S fraction, (**b**) $T_2$*-S value, (**c**) $T_2$*-S intensity, (**d**) $T_2$*-M fraction, (**e**) $T_2$*-M value, (**f**) $T_2$*-M intensity, (**g**) $T_2$*-L fraction, (**h**) $T_2$*-L value and (**i**) $T_2$*-L intensity.

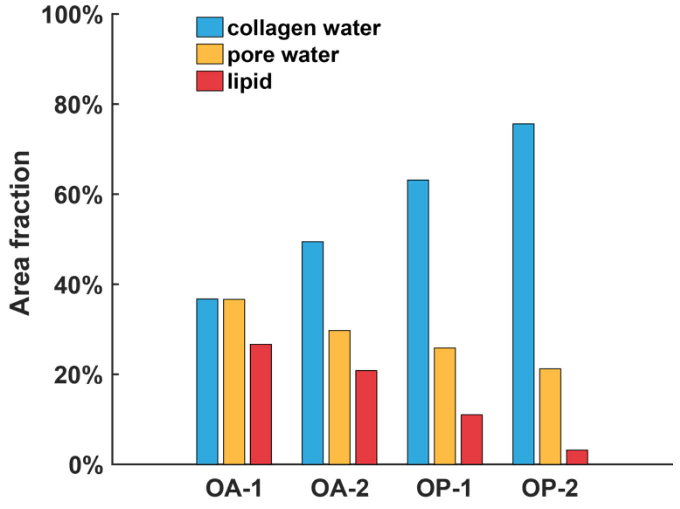


**Fig. S16 | The area fractions of different components in human bone samples**

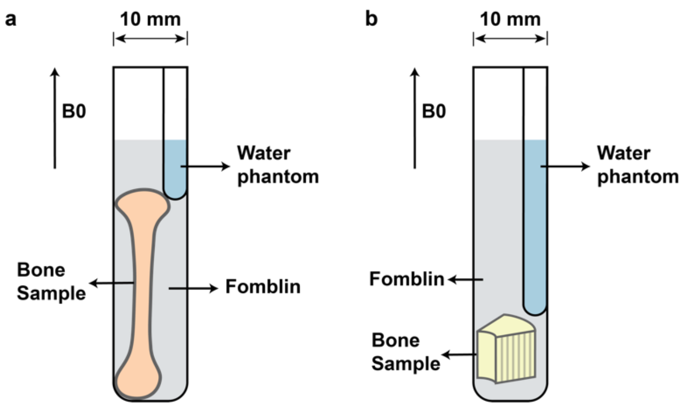


**Fig. S17 | MRI sample preparation.** (**a**) whole piece of rat femur and (**b**) extracted cortical tissue from large mammals in a 10-mm glass tube.

**Table S1 | The comparison of $T_2$* values of cortical bones**

| Cortical bone | Scanner | Field (T) | $T_2$* (ms) of bound water | $T_2$* (ms) of pore water | $T_2$* (ms) of lipids |
|---|---|---|---|---|---|
| Human[20] | Custom-built MRI | 0.94 | / | 1.1~2.7 (lacuna) 28~68 (canal) | / |
| Human[24] | GE Signa HDx | 3 | 0.24~0.32 | 1.9~2.5 | / |
| Human[26] | Siemens TIM Whole body | 3 | 0.29~0.38 | 1.6~5.3 | / |
| Bovine[30] | GE Signa TwinSpeed | 3 | 0.29~0.42 | 2.4~2.8 | / |
| Human[32] | GE Signa TwinSpeed | 3 | 0.37~0.41 | / | / |
| Mammals (this work) | Bruker micro-imaging | 14 | 0.1~0.5 | 1~4 | 4~8 |

**Table S2 | The comparison of MRI resolutions for bone studies**

| Bone samples | Scanner | Field (T) | FOV ($cm^2$ or $cm^3$) | Matrix Size | Resolution ($\mu m^2$ or $\mu m^3$) |
|---|---|---|---|---|---|
| Human[24] | GE Signa HDx | 3 | 8×8 | 256×256 | 313×313 |
| Human[25] | Siemens TIM Whole body | 3 | 18×18 | 512×512 | 352×352 |
| Human[26] | Siemens Whole body | 3 | 16×16×16 | 320×320×320 | 500×500×500 |
| Bovine[30] | GE Signa TwinSpeed | 3 | 8×8 | 256×256 | 313×313 |
| Human[32] | GE Signa TwinSpeed | 3 | 12×12 | 512×511 | 234×234 |
| Human (this work) | Bruker micro-imaging | 14 | 0.8×0.8×0.8 | 128×128×128 | 62.5×62.5×62.5 |

**Table S3 | The measurement results of micro-CT and UTE-MRI**

| Bone samples | Micro-CT Porosity (%) | BV/TV (%) | $T_2$*-S fraction (%) | $T_2$*-M fraction (%) | $T_2$*-L fraction (%) |
|---|---|---|---|---|---|
| Human | 22.2 | 77.8 | 41 | 24 | 35 |
| Sheep | 7.60 | 92.4 | 85 | 15 | 0 |

**Table S4 | Main parameters in the MR experiments.**

| Sequence | Parameter | Values |
|---|---|---|
| Direct excitation | 90° pulse length | 4–8 μs |
| | Power | 80 W |
| | TR | 5–6 s |
| Spin echo | TE | 8 μs–8 ms |
| UTE-3D | TE | 8 μs–8 ms |
| Normal resolution | Gradient strength (T/m) | 0.1957 |
| | TR | 30 ms |
| | Flip angle | 8.1° |
| UTE-3D | TE | 8 μs–8 ms |
| Ultra-resolution | Gradient strength (T/m) | 0.2936 (for human)<br>0.7040 (for sheep) |
| | TR | 50 ms |
| | Flip angle | 20° |
| MSME | Echo time | 2.6 ms–130 ms (50 echoes) |
| | Repetition time | 30 ms |
| | Flip angle | 90° (excitation), 180° (refocusing) |
